# Impact of confirmatory test results on subtype classification and biochemical outcome following unilateral adrenalectomy in patients with primary aldosteronism

**DOI:** 10.3389/fendo.2024.1495959

**Published:** 2024-11-29

**Authors:** Hediyeh Daneshpour, Denise Brüdgam, Isabel Stüfchen, Daniel Alexander Heinrich, Martin Bidlingmaier, Felix Beuschlein, Lydia Kürzinger, Tracy Ann Williams, Martin Reincke, Holger Schneider, Christian Adolf

**Affiliations:** ^1^ Medizinische Klinik und Poliklinik IV, Klinikum der Universität München, Ludwig-Maximilians-Universität (LMU) München, Munich, Germany; ^2^ Klinik für Endokrinologie, Diabetologie und Klinische Ernährung, Universitätsspital Zürich (USZ) und Universität Zürich (UZH), Zurich, Switzerland; ^3^ The LOOP Zurich - Medical Research Center, Zurich, Switzerland; ^4^ Department of Internal Medicine I, Division of Endocrinology and Diabetes, University Hospital of Würzburg, Würzburg, Germany

**Keywords:** aldosterone, confirmatory test, saline infusion test, captopril challenge test, PASO criteria

## Abstract

**Context:**

Primary aldosteronism (PA) is the most common form of endocrine hypertension. According to the Endocrine Society Practice Guidelines, the diagnosis of PA requires a pathological screening test result and non-suppressible aldosterone levels during confirmatory testing. Sequential testing with more than one confirmatory test may result in discordant test results.

**Objective and patients:**

We investigated the association of discordant results of captopril challenge test (CCT) and saline infusion test (SIT) on patient subtype classification by adrenal vein sampling (AVS) and outcome in 111 consecutive patients from the German Conn’s Registry. Concordance was defined as non-suppressible aldosterone levels upon both tests, while discordance was defined as conflicting test results. Patients with unilateral disease were offered adrenalectomy (ADX). Biochemical and clinical outcomes were assessed using the PASO criteria.

**Results:**

85 of 111 (77%) patients had concordant results of CCT and SIT. Although baseline characteristics were comparable between patients with concordant and discordant tests, the latter had significantly lower aldosterone levels after testing (CCT: 170 vs. 114pg/ml; SIT: 139 vs. 101pg/ml; p=0.004). In 35% of patients with discordant (n=9) and 46% of concordant test results (n=39), AVS suggested lateralized PA. In 36 of 48 cases ADX was performed. 86% of patients with discordant and 72% with concordant results had complete biochemical success.

**Conclusion:**

The use of two confirmatory tests in patients with PA results in discordant results in approximately 23% of cases. Patients having discordant confirmatory test results had a comparable rate of lateralized PA and underwent adrenalectomy with similar long-term outcome.

## Introduction

Primary aldosteronism (PA) is the most frequent cause of endocrine arterial hypertension and affects about 5-15% of hypertensive patients (1, 2). Patients with PA have a higher risk for cardiovascular complications such as stroke and myocardial infarction as well as metabolic diseases like diabetes mellitus type 2, compared to matched patients with essential hypertension (3–7). The diagnosis is often protracted due to challenges including adjustment of antihypertensive medication before screening and confirmation testing (8, 9). The fact that only 1% of patients with treatment-resistant hypertension undergo guideline-recommended screening for PA underlines the importance of increasing the awareness for PA, as the early diagnosis is essential to reduce cardiovascular risks (10, 11).

The Clinical Practice Guideline for PA recommends screening by measuring the plasma aldosterone to renin ratio (ARR) with adjusted medication to identify potential cases of PA. Whereas patients with a strong biochemical phenotype can directly bypass to adrenal vein sampling (AVS) according to Clinical Practice Guidelines, patients with a pathological ARR usually undergo at least one confirmatory test to ensure or reject the diagnosis (9, 12, 13). In clinical routine either the saline infusion test (SIT) or the captopril challenge test (CCT) is performed. Because of the high variability of aldosterone and confirmatory tests, there might be discordant results in SIT and CCT (14, 15). Considering limited sensitivity and specificity, the Japanese Endocrine Society Guideline recommends performing at least two confirmatory tests, regardless of the result of the first one to make a definitive diagnosis (16).

In this context, in 2021 Fukumoto et al. reported that most of the patients with a pathological result in only one confirmatory test (discordant) had milder forms of PA indicated by 96% of patients showing a non-lateralized disease (17). Populations from Asian countries exhibit differences in the relative proportions of each PA subtype and of mutation status in aldosterone-producing adenomas, which predominantly cause unilateral PA (16, 18). These factors can influence the results of confirmatory tests. We investigated patients with diagnosed PA from the Munich Center of the German Conn’s Registry who underwent two confirmatory tests (CCT and SIT) on subtyping and outcome of PA.

## Subjects and methods

### Patients

**Figure 1:**
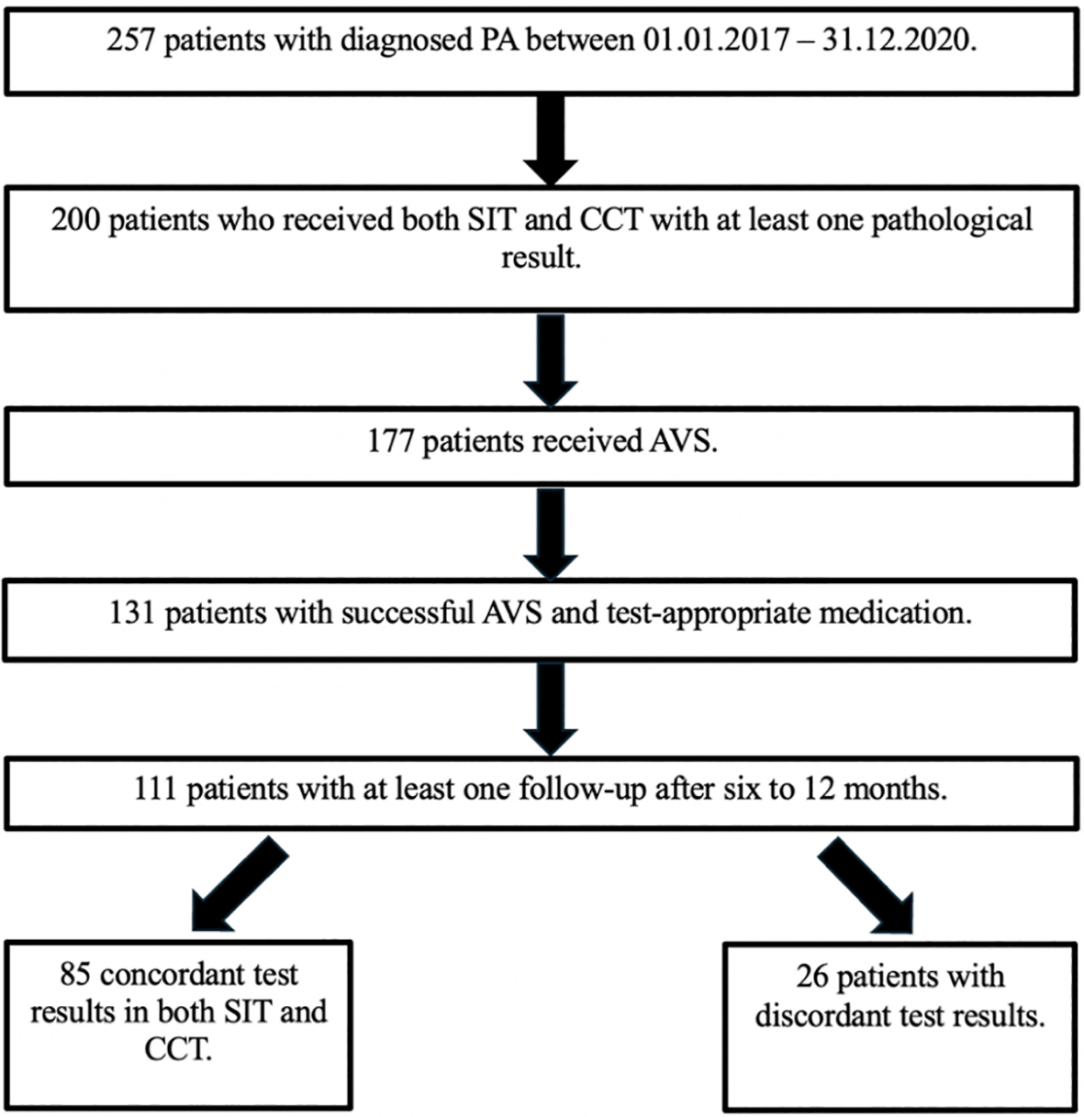
Patient flowchart. AVS, adrenal vein sampling; CCT, captopril challenge test; PA, primary aldosteronism; SIT, saline infusion test.

Between 2017 and 2020, 257 consecutive patients were diagnosed with PA in the Munich center of the German Conn’s Registry. All patients were supposed to undergo SIT as well as CCT. Due to Covid-19 pandemic and long traveling distances, only 200 patients underwent both confirmatory tests and had a pathological result in at least one of them. Of the latter, 177 underwent AVS with 131 patients showing successful AVS and receiving appropriate medication with limited impact on the ARR according to the Endocrine Society Guidelines (9). Thereof, 111 patients had at least one follow-up after six to 12 months, which represents the cohort included in the study (**Figure 1**).

### Study protocol

#### Screening and confirmation tests

All patients were screened according to the Endocrine Society Guideline (9) using the ARR (cut-off 12.0 pg/ml/μU/ml, sitting position),with a cut-off of at least 60 pg/ml for PAC (19). Before screening or confirmatory testing, antihypertensive medication was withdrawn for at least one or four weeks (contraceptives, mineralocorticoid receptor antagonists). If not possible, patients received medication with limited impact on the ARR, such as the alpha 1-adrenergic receptor antagonist doxazosin or the calcium-channel antagonist verapamil (9). Patients with pathological ARR ≥ 12 pg/ml/μU/ml underwent CCT and SIT independently of the results of the first test. Both tests were performed in a seated position as this measurement shows superiority to the recumbent position (20).

For the SIT, 2000 ml sodium chloride solution (0.9%) was applied by infusion in a seated position over a duration of 4 hours in the morning (8-12 am). Blood samples were drawn before (0h) and after infusion (4h). During the whole test the patients were monitored. SIT was considered pathological if aldosterone value after testing was ≥ 60 pg/ml and baseline ARR was ≥ 12 pg/ml/μU/ml (9).

For the CCT, blood samples were drawn after the patient was resting for 30 min followed by the oral administration of 50 mg captopril. After 60 min and 120 min blood was collected again. If the aldosterone was suppressed less than 30% of baseline value with a baseline ARR ≥ 12 pg/ml/μU/ml, PA was diagnosed (9).

For assessing cortisol co-secretion, a low-dose dexamethasone suppression test was performed by oral administration of 1 mg dexamethasone at 11 pm. Blood was collected at 8 am the next day. Cortisol co-secretion was diagnosed if the cortisol concentration was equal to or above 1.8µg/dl.

#### Classification of patients

Patients were classified as concordant if both confirmatory tests (SIT and CCT) showed a pathological result with non-suppressible aldosterone. If only one confirmatory test showed a pathological result, they were classified as discordant.

#### Subtyping and outcome assessment

After diagnosis of PA all patients underwent further subtyping with AVS as previously reported (21). Successful cannulation of the adrenal vein was assumed when the selectivity index (SI), which is defined as the measured plasma cortisol concentration in the respective adrenal vein to the peripheral vein, was ≥ 2.0. For patients with a lateralization index ≥ 4 unilateral disease was assumed and unilateral ADX was offered. Patients without lateralization, those who refused surgery or had contraindications for surgery received medical treatment with mineralocorticoid receptor antagonists (MRA).

All patients were re-evaluated approximately six to 12 months after initiation of specific treatment by ADX or MRA. The clinical and biochemical outcome was assessed according to the PASO criteria six to 12 months after surgery (22).

#### Laboratory analysis

Blood collection was performed standardized between 8 am and 10 am in a fasting state after a rest of 15 minutes in a seated position at baseline and at follow-up. Samples were either analyzed directly or processed and stored at -80°C until analysis.

Aldosterone, renin and cortisol were measured using the Diasorin Liaison CLIA. All other analyses were performed in our central laboratory using standard methods.

### Statistical analysis

Data between groups was compared using Mann-Whitney U test or chi-square test for numerical or categorical variable, respectively. In this study, continuous variables are reported as mean values with ± standard deviation (SD), while categorical variables are presented as frequencies and percentage. Two-tailed probability values of <5% were considered to be statistically significant. Statistical analysis was performed using standard statistical software (IBM SPSS Statistics for Windows, Version 25. Armonk, NY: IBM Corp.).

## Results

### Clinical and biochemical characteristics of the total cohort

Out of 200 patients with PA who underwent both confirmatory tests and had a pathological result in at least one of them, 111 were included in the study. The mean age was 50 ± 11 years and the mean BMI was 27.4 ± 4.7 kg/m². On average, PA was diagnosed 8 years after the known onset of hypertension. In total, a higher proportion of men were diagnosed with PA than women (male: n=60; 54.1%; female: n=51; 45.9%). Besides plasma aldosterone concentration (193 pg/ml), mean systolic (SBP) and diastolic (DBP) blood pressure levels were still elevated despite a defined daily dose of antihypertensives (DDD) of 1.9 (SBP 155 mmHg; DBP 97 mmHg).

### Characteristics of patients with concordant and discordant confirmatory tests

Out of the total cohort 77% (n=85) showed concordant and 23% (n=26) discordant results in SIT and CCT. 15 of these patients showed a positive test result in SIT and 11 in CCT (**Table 1**). Patients with concordant and discordant tests were comparable for baseline characteristics such as gender, age, duration of hypertension, BMI, plasma aldosterone and renin concentration. Although systolic blood pressure was comparable, diastolic blood pressure was significantly higher in patients with discordant test results (95 vs. 102 mmHg; p=0.015).

**Table 1:** Characteristics of patients with concordant and discordant confirmatory tests at baseline.

Patient characteristics	n	Concordant	n	Discordant	P	n	Total cohort
		Before treatment		Before treatment			Before treatment
Sex [f/m]	85	40/45	26	11/15	0.671	111	51/60
Age	85	51.1 ± 10.8	26	47.8 ± 11.4	0.256	111	50.3 ± 11
Duration of hypertension [months]	85	105 ± 98	25	71 ± 78	0.133	110	97 ± 95
BMI [kg/m²]	85	27.2 ± 4.5	26	27.7 ± 5.8	0.870	111	27.4 ± 4.7
SBP [mmHg]	85	154 ± 16	26	160 ± 22	0.245	111	155 ± 18
DBP [mmHg]	85	95 ± 11	26	102 ± 12	**0.015**	111	97 ± 12
Serum potassium [mmol/l]	85	3.6 ± 0.4	26	3.8 ± 0.4	0.228	111	3.7 ± 0.4
PAC [pg/ml]	85	197 ± 140	26	178 ± 128	0.298	111	193 ± 137
DRC [µU/ml]	85	3.9 ± 3.4	26	4.1 ± 2.9	0.841	111	3.9 ± 3.3
ARR [pg/ml/μU/ml]	85	68.5 ± 55.0	26	53.1 ± 35.9	0.144	111	64.9 ± 51.5
DDD [n]	85	1.9 ± 2.1	26	1.8 ± 1.7	0.839	111	1.9 ± 2.0
Post DST result [mg/dl]	60	1.8 ± 1.4	20	1.3 ± 0.4	0.316	80	1.7 ± 1.2

ARR, aldosterone-to-renin ratio; BMI, body mass index; DBP, diastolic blood pressure; DDD, defined daily doses of antihypertensive medications; DRC, direct renin concentration; DST, dexamethasone suppression test; PAC, plasma aldosterone concentration; SBP, systolic blood pressure.

Significance is marked bold.

However, aldosterone was less suppressible in both tests in the patient group with concordant test results in comparison to patients with discordant test results (SIT: 139 vs. 101 pg/ml; p= 0.014; CCT: 170 vs. 114 pg/ml; p=0.004) and renin levels tended to be lower at baseline (2.7 vs. 4.0 µU/ml; p= 0.018). After CCT, the discordant patient group featured a more pronounced raise in renin (3.3 vs. 4.9 µU/ml; p=0.010).

There was no difference concerning cortisol co-secretion between patients with concordant and discordant test results (1.8 vs. 1.3 mg/dl; p=0.316).

47% (n=40) of patients with concordant and 46% (n=12) with discordant test results revealed nodules of the adrenal gland in imaging (p=0.936). Patients with concordant test results tended to have larger nodules (17 vs. 13mm; p=0.061) (**Table 2**).

**Table 2:** Characteristics of patients with concordant and discordant confirmatory tests according to subtype diagnosis.

Subtyping	n	Concordant	n	Discordant	P
Presence of nodule [yes/no]	85	40/45	26	12/14	0.936
Size of largest nodule at imaging [mm]	38	17 ± 8	11	13 ± 6	0.061
Subtype according to AVS [unilateral/bilateral]	85	39/46	26	9/17	0.310
Lateralization index [n]	85	11.0 ± 18.4	26	7.8 ± 12.3	0.648
Contralateral suppression according to AVS [yes/no]	39	33/6	9	6/3	0.214

AVS, adrenal vein sampling.

According to AVS subtyping, 54% (n=46) of patients with concordant and 65% (n=17) with discordant test results showed a non-lateralized disease. 46% of patients in the group with concordant (n=39) and 35% with discordant results (n=9) were lateralized (p=0.310).

### Characteristics of patients with concordant and discordant confirmatory tests according to subtype diagnosis

Considering patients with non-lateralized disease, the ARR was significantly higher in patients with concordant confirmatory test results at baseline (60.9 vs. 42.5 pg/ml/μU/ml; p=0.037), whereas other parameters were comparable in both groups (**Table 3**). In confirmatory testing, renin was significantly lower in the group with concordant results before (4.3 vs. 2.5 µU/ml; p=0.013) and after CCT (4.6 vs. 3.0 µU/ml; p=0.041). The drop of aldosterone in CCT was also significantly smaller in patients with concordant testing (5 vs. 24%; p=0.003). Patients with discordant and concordant confirmatory tests with unilateral disease did not differ significantly in baseline characteristics, but patients with discordant test results had stronger suppression during confirmatory test by SIT and CCT compared to those with concordant confirmatory tests (SIT: 22 vs. 36%; p=0.023; CCT: 3 vs. 29%; p=0.008).

**Table 3:** Characteristics of bilateral and unilateral patients with concordant and discordant confirmatory tests.

Patient characteristics	Non-lateralized PA (n=63)					Lateralized PA (n=48)				
	n	Concordant	n	Discordant	P	n	Concordant	n	Discordant	P
		Before treatment		Before treatment			Before treatment		Before treatment	
Sex [f/m]	46	20/26	17	7/10	0.870	39	20/19	9	4/5	0.712
Age	46	52.7 ± 10.2	17	47.5 ± 11.2	0.152	39	49.2 ± 11.3	9	48.3 ± 12.4	0.959
Duration of hypertension [months]	46	120 ± 107	16	74 ± 80	0.122	39	88 ± 86	9	66 ± 78	0.419
BMI [kg/m²]	46	28.5 ± 4.8	17	28.5 ± 6.4	0.467	39	25.7 ± 3.4	9	26.6 ± 4.7	0.585
SBP [mmHg]	46	156 ± 17	17	163 ± 24	0.430	39	152 ± 15	9	155 ± 18	0.637
DBP [mmHg]	46	96 ± 13	17	104 ± 11	**0.013**	39	96 ± 9	9	99 ± 13	0.514
Serum potassium [mmol/l]	46	3.7 ± 0.3	17	3.8 ± 0.4	0.454	39	3.5 ± 0.5	9	3.6 ± 0.5	0.453
PAC [pg/ml]	46	171 ± 77	17	170 ± 144	0.249	39	227 ± 187	9	193 ± 99	0.937
DRC [µU/ml]	46	3.6 ± 2.7	17	4.7 ± 3.3	0.347	39	4.3 ± 4.2	9	2.8 ± 1.5	0.432
ARR [pg/ml/μU/ml]	46	60.9 ± 37.1	17	42.5 ± 28.3	**0.037**	39	77.4 ± 70.1	9	73.3 ± 41.7	0.606
DDD [n]	46	1.7 ± 1.3	17	1.7 ± 1.8	0.595	39	2.1 ± 2.7	9	2.0 ± 1.6	0.661

ARR, aldosterone-to-renin ratio; BMI, body mass index; DBP, diastolic blood pressure; DDD, defined daily doses of antihypertensive medications; DRC, direct renin concentration; PAC, plasma aldosterone concentration; SBP, systolic blood pressure.

Significance is marked bold.

### Clinical and biochemical outcome of patients treated by MRA or ADX

Neither patients treated with MRA (n=75) nor patients after ADX showed differences in baseline plasma aldosterone, renin and potassium when comparing the cohort with concordant and discordant test results in follow-up visit (**Table 4**).

**Table 4:** Characteristics of MRA and ADX treated patients in follow-up visit.

Patient characteristics	MRA treated patients (n=75)				P	AXD treated patients (n=36)				P
	n	Concordant	n	Discordant		n	Concordant	n	Discordant	
SBP [mmHg]	56	133 ± 14	19	139 ± 16	0.265	29	135 ± 14	7	129 ± 13	0.456
DBP [mmHg]	56	86 ± 9	19	92 ± 12	**0.014**	29	89 ± 10	7	87 ± 11	0.557
Serum potassium [mmol/l]	56	4.5 ± 0.4	19	4.3 ± 0.4	0.091	29	4.4 ± 0.3	7	4.6 ± 0.7	0.557
PAC [pg/ml]	56	306 ± 149	19	263 ± 195	0.080	29	102 ± 74	7	81 ± 39	0.480
DRC [µU/ml]	56	49.4 ± 121.1	19	25.7 ± 37.9	0.377	29	13.6 ± 16.2	7	16.0 ± 14.6	0.584
ARR [pg/ml/μU/ml]	56	32.3 ± 43.9	19	24.2 ± 21.1	0.719	29	19.4 ± 21.3	7	16.7 ± 24.0	0.410
DDD [n]	56	2.2 ± 2.2	19	2.2 ± 2.5	0.760	29	0.64 ± 1.1	7	1.3 ± 1.6	0.433
MRA DDD	56	0.7 ± 0.8	19	0.6 ± 0.3	0.429					

ARR, aldosterone-to-renin ratio; BMI, body mass index; DBP, diastolic blood pressure; DDD, defined daily doses of antihypertensive medications; DRC, direct renin concentration; MRA, mineralocorticoid receptor antagonist; PAC, plasma aldosterone concentration; SBP, systolic blood pressure.

Significance is marked bold.

Of 48 patients with unilateral disease, 36 underwent ADX. Tumor tissue was evaluated for PA-driver mutations in 21 patients. Almost 50% of adenomas (n=10) carried a KCNJ5 mutation, of which 90% (n=9) were female. 90% (n=9) of all patients with KCNJ5 mutation had concordant results in confirmatory testing.

**Figure 2:**
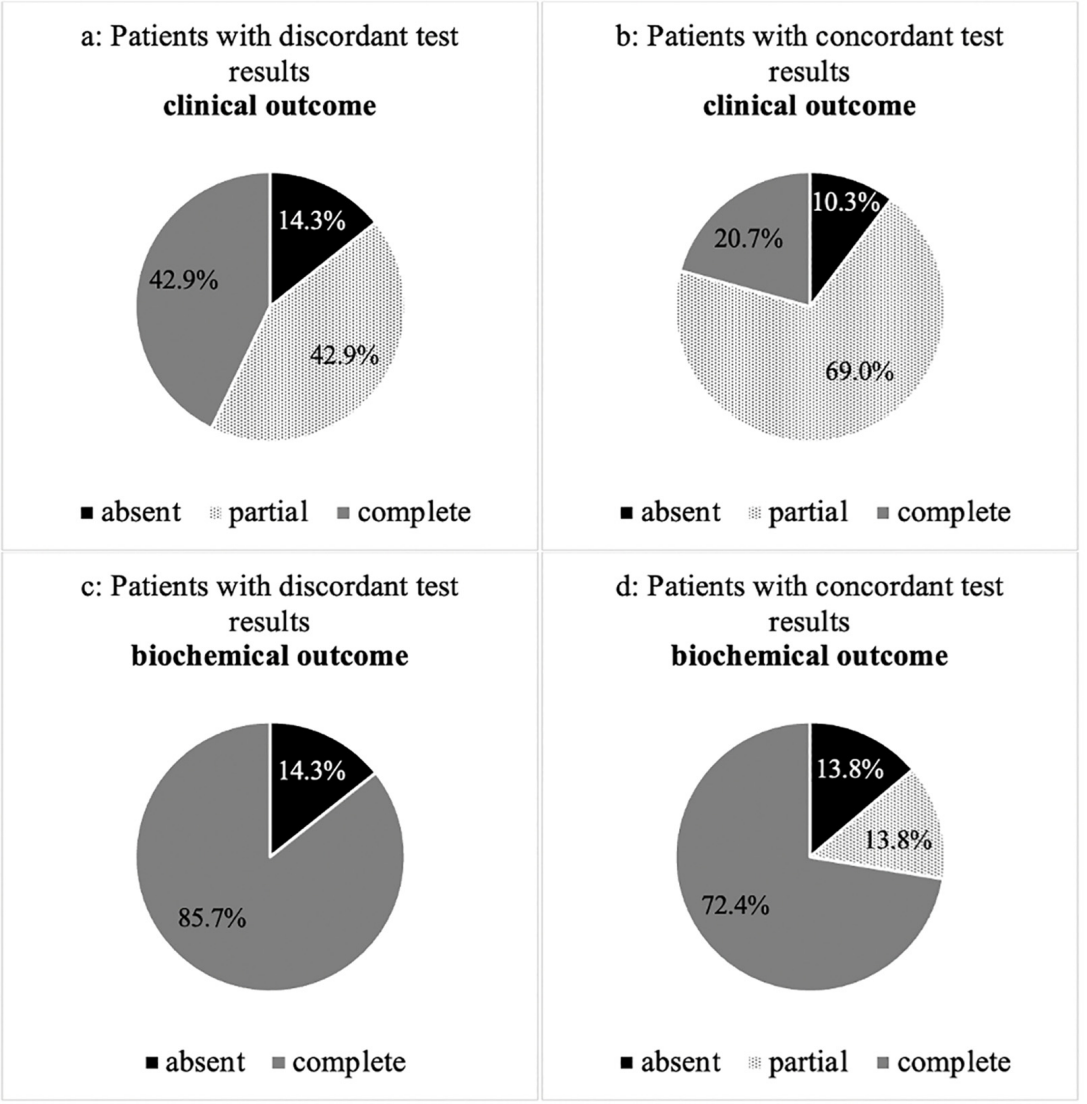
**(A–D)** Clinical and biochemical outcome of patients with concordant and discordant test results after adrenalectomy according to PASO criteria.

We evaluated the biochemical and clinical outcome of patients with concordant (n=29) and discordant (n=7) test results after ADX according to the PASO criteria (19). Overall, 72% (n=21) of patients with concordant and 86% (n=6) with discordant confirmatory tests had a complete biochemical remission (p=0.375). 14% (n=14) of the group with concordant results revealed a partial biochemical remission, whereas none of those with discordant tests showed this result (p=0.297). 14% (n=4) of patients with concordant and 14% (n=1) with discordant confirmatory tests had an absent biochemical remission (p=0.851) (**Figure 2**).

Considering the clinical outcome, 69% (n=20) of the group with concordant and 43% (n=3) with discordant results had a complete clinical remission (p=0.224). 21% (n=6) with concordant and 43% (n=3) with discordant testing revealed a partial clinical remission (p=0.270). 10% (n=3) of patients with concordant and 14% (n=1) with discordant results had an absent clinical remission (p=0.973).

### Characteristics of unilateral patients with discordant confirmatory tests and pathology in either SIT or CCT

Despite significantly higher plasma aldosterone concentration (PAC) values in patients with pathological SIT results (90 vs. 240 pg/ml; p=0.024), there were no significant differences in baseline characteristics when splitting patients with unilateral disease and discordant confirmatory tests into subgroups of patients with pathological SIT or CCT.

Considering subtyping, 6 out of 11 patients with pathology in CCT and 6 out of 15 patients with pathology in SIT revealed nodules in imaging (p=0.462). Patients with pathological results in CCT tended to show larger nodules than patients with pathological SIT, even if the difference did not reach statistical significance (12.0 vs. 7.0 mm; p=0.333).

In AVS, there was no difference between patients with pathology in CCT (8 out of 11 were non-lateralized; 72%) and patients with pathology in SIT (9 out of 15; 60%) (p=0.500).

## Discussion

In clinical routine PA is highly underdiagnosed due to the complex diagnostic work up (10, 23). The present study adds further evidence that confirmatory tests lack sensitivity, even if performed properly (14, 24, 25).

In this study almost one fourth (23%) of patients with PA would have been missed, if only one confirmatory test had been performed. In this context, it is of particular importance that this would also negatively impact patients with non-lateralized PA with a strong clinical and biochemical phenotype, as these patients can often undergo surgery to achieve biochemical cure.

To improve the diagnostic procedure and to reduce the burden of utilized healthcare resources, machine learning-based models and steroid metabolomics-driven diagnostic strategies are currently developed (26, 27). Whereas for patients with strong biochemical and clinical phenotype Clinical Practice Guidelines offer the possibility to bypass the confirmatory tests, this pathway is not recommended for milder cases of PA, although this topic is also controversially discussed (12, 28).

In the present study patients with concordant and discordant test results were comparable for gender, age, biochemical and clinical phenotype and could hardly be divided from each other at first presentation. In addition, the AVS lateralization index was not different between patients with concordant and discordant test results (11.0 vs. 7.8; p= 0.648), as well as between patients with or without cortisol co-secretion (p=0.886), whose impact on AVS parameters is still under debate (29, 30).

Interestingly, one third of discordant patients showed a unilateral subtype, and outcome after surgery in patients diagnosed with unilateral disease was not different between patients with discordant and concordant test results. Moreover, there was no evidence in favor of either confirmatory test for the diagnosis of PA or in predicting either lateralization or outcome of the disease (14).

The results of our present study are in contrast to the findings of Fukumoto et al. who reported that 96% of all discordant and 57% of all patients with concordant confirmatory tests were diagnosed with non-lateralized PA, resulting in the conclusion that discordant patients could consequently be treated with MRA without further testing (17).

In our current cohort, lateralized disease was driven by a KCNJ5-mutated APA in 50% of patients, which is comparable with other studies. Interestingly, most patients with APA-KCNJ5 mutations had concordant test results, which could explain the differences compared to the Fukumoto group as there is a much higher frequency of KCNJ5 mutation (over 80%) in patients with lateralized disease in Asian countries (17).

Based on these findings, further testing can be reasonable in Western countries if patients with highly suspected PA cannot bypass confirmatory testing. However, our findings do not support the routine implementation of two confirmatory tests due to the logistical challenges and the substantial burden of utilized healthcare resources.

As evidence suggests an ongoing increased cardiovascular risk despite MRA treatment - at least if renin levels are suppressed (31–33) - discordant test results should not be used to withhold AVS testing. This is further underlined by the fact that the biochemical and clinical outcome were comparable between patients with concordant and discordant confirmatory test results.

We acknowledge the limitation that the measurements in this study were performed with immunoassay. Compared to the commercial immunoassay, the LC-MS/MS seems to show more accurate results and is considered to be more reliable (34, 35). Although LC-MS/MS performs superior in terms of sensitivity and specificity, it must be pointed out that it tends to show lower values compared to immunoassay, which calls for specifically established reference intervals for this particular method. Moreover, a relevant number of patients were excluded because of our strict inclusion criteria. Due to the small sample size of the cohort included, no definitive conclusion can be drawn. Therefore, more investigation is necessary to address this issue. The strengths of our study include a structured and detailed patient phenotyping of the homogeneously characterized study population including data on AVS and mutation status.

In conclusion, our data show that the combined use of SIT and CCT in patients with PA results in discordant results in one fourth of cases. Patients with discordant confirmatory test results had a comparable rate of lateralized PA and underwent adrenalectomy with similar long-term outcome. Thus, further improvements in the diagnostic workup are necessary.

## Data Availability

The original contributions presented in the study are included in the article/**Supplementary Material**. Further inquiries can be directed to the corresponding author.
