# Supplementary material for: Impact of confirmatory test results on subtype classification and biochemical outcome following unilateral adrenalectomy in patients with primary aldosteronism
Source: Front Endocrinol (Lausanne). 2024 Nov 29;15:1495959. doi: 10.3389/fendo.2024.1495959 (PMC11637841; doi:10.3389/fendo.2024.1495959)
Supplement: Supplementary file 1 [file DataSheet1.docx]

Supplementary Table 1. Values before and after confirmatory testing.

| **Confirmatory Tests** | **n** | **Concordant** | **n** | **Discordant** | **P** |
| --- | --- | --- | --- | --- | --- |
| PAC before SIT [pg/ml] | 85 | 198 ± 139 | 26 | 173 ± 106 | 0.365 |
| DRC before SIT [µU/ml] | 85 | 3.9 ± 3.4 | 26 | 4.8 ± 4.6 | 0.736 |
| PAC after SIT [pg/ml] | 85 | 139 ± 101 | 26 | 101 ± 57 | **0.014** |
| DRC after SIT [µU/ml] | 85 | 2.7 ± 1.6 | 26 | 3.1 ± 2.1 | 0.158 |
|  |  |  |  |  |  |
| PAC before CCT [pg/ml] | 85 | 177 ± 100 | 26 | 166 ± 88 | 0.738 |
| DRC before CCT [µU/ml] | 85 | 2.7 ± 1.8 | 26 | 4.0 ± 3.7 | **0.018** |
| PAC after CCT [pg/ml] | 85 | 170 ± 101 | 26 | 114 ± 49 | **0.004** |
| DRC after CCT [µU/ml] | 85 | 3.3 ± 3.0 | 26 | 4.9 ± 5.1 | **0.010** |
| ARR SIT [pg/ml/μU/ml] | 85 | 69.2 ± 54.7 | 26 | 49.9 ± 33.2 | 0.058 |
| ARR CCT [pg/ml/μU/ml] | 85 | 78.4 ± 54.4 | 26 | 53.6 ± 24.8 | **0.044** |
| SIT Delta Aldo [%] | 85 | -27.0 ± 20.5 | 26 | -35.9 ± 21.3 | **0.010** |
| SIT Delta Renin [%] | 85 | -16.8 ± 27.1 | 26 | -18.9 ± 25.4 | 0.927 |
| CCT Delta Aldo [%] | 85 | -3.9 ± 19.5 | 26 | -25.9 ± 21.5 | **0.000** |
| CCT Delta Renin [%] | 85 | 18.3 ± 50.2 | 26 | 22.6 ± 44.6 | 0.279 |

Abbreviations: ARR, aldosterone-to-renin ratio; CCT, captopril challenge test; DRC, direct renin concentration; PAC, plasma aldosterone concentration; SIT, saline infusion test.

Significance is marked bold.

Supplementary Table 2. Confirmatory test values of concordant and discordant patients with bilateral and unilateral PA.

|  | **Non-Lateralized PA** | | | |  | **Lateralized PA** | |  |  |  |
| --- | --- | --- | --- | --- | --- | --- | --- | --- | --- | --- |
| **Confirmatory Tests** | **n** | **Concordant** | **n** | **Discordant** | **P** | **n** | **Concordant** | **n** | **Discordant** | **P** |
| PAC before SIT [pg/ml] | 46 | 175 ± 74 | 17 | 170 ± 117 | 0.403 | 39 | 226 ± 187 | 9 | 180 ± 89 | 0.874 |
| DRC before SIT [µU/ml] | 46 | 3.6 ± 2.7 | 17 | 5.5 ± 5.3 | 0.260 | 39 | 4.3 ± 4.2 | 9 | 3.3 ± 2.4 | 0.305 |
| PAC after SIT [pg/ml] | 46 | 117 ± 57 | 17 | 99 ± 62 | 0.111 | 39 | 166 ± 131 | 9 | 106 ± 50 | 0.086 |
| DRC after SIT [µU/ml] | 46 | 2.6 ± 1.6 | 17 | 3.3 ± 2.4 | 0.137 | 39 | 2.8 ± 1.7 | 9 | 2.7 ± 1.2 | 0.736 |
| PAC before CCT [pg/ml] | 46 | 154 ± 66 | 17 | 160 ± 93 | 0.944 | 39 | 206 ± 125 | 9 | 179 ± 81 | 0.704 |
| DRC before CCT [µU/ml] | 46 | 2.5 ± 1.1 | 17 | 4.3 ± 3.8 | **0.013** | 39 | 3.0 ± 2.3 | 9 | 3.5 ± 3.5 | 0.751 |
| PAC after CCT [pg/ml] | 46 | 143 ± 63 | 17 | 113 ± 53 | **0.050** | 39 | 202 ± 127 | 9 | 116 ± 42 | 0.052 |
| DRC after CCT [µU/ml] | 46 | 3.0 ± 1.8 | 17 | 4.6 ± 3.7 | **0.041** | 39 | 3.7 ± 4.0 | 9 | 5.4 ± 7.2 | 0.139 |
| ARR SIT [pg/ml/μU/ml] | 46 | 62.9 ± 37.0 | 17 | 41.3 ± 23.8 | **0.021** | 39 | 76.6 ± 70.0 | 9 | 66.2 ± 43.1 | 0.938 |
| ARR CCT [pg/ml/μU/ml] | 46 | 68.3 ± 35.1 | 17 | 48.3 ± 25.5 | **0.047** | 39 | 90.3 ± 69.4 | 9 | 63.4 ± 21.2 | 0.659 |
| SIT Delta Aldo [%] | 46 | -31.4 ± 18.3 | 17 | -35.6 ± 21.0 | 0.233 | 39 | -21.9 ± 22.0 | 9 | -36.5 ± 23.2 | **0.023** |
| SIT Delta Renin [%] | 46 | -17.6 ± 25.3 | 17 | -25.0 ± 26.3 | 0.510 | 39 | -15.8 ± 29.3 | 9 | -7.4 ± 20.1 | 0.209 |
| CCT Delta Aldo [%] | 46 | -5.0 ± 20.8 | 17 | -24.1 ± 18.9 | **0.003** | 39 | -2.6 ± 18.1 | 9 | -29.4 ± 26.6 | **0.008** |
| CCT Delta Renin [%] | 46 | 22.4 ± 59.3 | 17 | 16.7 ± 45.8 | 0.986 | 39 | 13.5 ± 36.9 | 9 | 33.5 ± 42.6 | 0.108 |

Abbreviations: ARR, aldosterone-to-renin ratio; CCT, captopril challenge test; DRC, direct renin concentration; PA, primary aldosteronism; PAC, plasma aldosterone concentration; SIT, saline infusion test.

Significance is marked bold.
